# TRIM52 inhibits Japanese Encephalitis Virus replication by degrading the viral NS2A

**DOI:** 10.1038/srep33698

**Published:** 2016-09-26

**Authors:** Wenchun Fan, Mengge Wu, Suhong Qian, Yun Zhou, Huanchun Chen, Xiangmin Li, Ping Qian

**Affiliations:** 1 State Key Laboratory of Agriculture Microbiology, Huazhong Agricultural University, Wuhan 430070, P. R. China; 2 Division of Animal Infectious Diseases, College of Veterinary Medicine, Huazhong Agricultural University, Wuhan 430070, China; 3 The Cooperative Innovation Center for Sustainable Pig Production, Huazhong Agricultural University Wuhan 430070, China

## Abstract

The members of tripartite-motif containing (TRIM) protein participate in various cellular processes and play an important role in host antiviral function. TRIM proteins exert their antiviral activity either directly by degrading viral proteins through their E3 ligase activity, or indirectly by promoting host innate immunity. This study demonstrated for the first time that TRIM52 is a novel antiviral TRIM protein against Japanese encephalitis virus (JEV) infection. Overexpression of TRIM52 restricted JEV replication in BHK-21 and 293T cells. In addition, JEV nonstructural protein 2A (NS2A) is a protein that interacts with TRIM52. Their interaction degraded NS2A in a proteasome-dependent manner via the E3 ligase activity of TRIM52. Thus, TRIM52 is a novel antiviral TRIM protein, and it exerted antiviral activity against JEV infection by targeting and degrading viral NS2A.

More than 70 distinct tripartite-motif containing (TRIM) proteins have been identified in humans and mice^1^,^2^. Members of the TRIM family proteins share a conserved domain architecture known as RBCC motif, which displays a highly conserved order that consists of N-terminus: a RING finger domain, one or two B-boxes domains, and a coiled-coil region^2^,^3^,^4^. The RING domain endues TRIM proteins with the function of E3-ubiquitin ligase, which can mediate the conjugation of proteins with ubiquitin, with small ubiquitin-like molecule modifier (SUMO) or with the ubiquitin-like molecule interferon (IFN) stimulated gene (ISG) of 15 kDa (ISG15). The CC region is associated with the formation of high molecular weight complexes formed between TRIM proteins. The C-terminal domains of TRIM proteins are variable, these domains include PRY/SPRY (B30.2), NHL repeats (NHL), COS box motif (COS), fibronectin type III motif (FN3), and uncharacterized motif^1^,^3^,^4^. The C-terminus often serves TRIM protein as a protein-protein interaction and/or RNA binding module^5^.

TRIM proteins are implicated in a variety of cellular functions, such as cell growth, differentiation, oncogenesis, inflammation, apoptosis, and innate antiviral immunity^1^,^2^,^3^,^6^,^7^,^8^,^9^,^10^,^11^. Accumulating studies have expounded on the important role of TRIM proteins in host responses to viral infections. These proteins exert their antiviral functions either directly or by regulating the innate antiviral immunity signaling of the host. Some TRIM proteins target innate immune pattern-recognition receptors to regulate host innate immune response^12^. TRIM25, is a famous TRIM protein, that promotes ubiquitination and activation of retinoic-acid-inducible gene-I (RIG-I) to sense RNA virus infection and induce IFN production to elicit host antiviral innate immunity^13^. TRIM9 short isoform was identified as a positive regulator in production of type I IFN, which serves as a platform that bridges GSK3β to TANK-binding kinase 1 (TBK1), resulting in activation of IRF3 signaling^14^. Ran discovered that ubiquitinated TRIM26 is associated with NF-κB essential modulator (NEMO) and promotes TBK1-NEMO interaction to activate TBK1^15^. Moreover, some TRIM proteins exert direct antiviral activity. TRIM22 restricts influenza A virus (IAV) and hepatitis C virus (HCV) infection by targeting and degrading IAV viral nucleoprotein (NP) and HCV viral NS5A in a proteasome-dependent manner^16^,^17^. TRIM32 also exerts antiviral effect against IAV through ubiquitination of PB1 polymerase^18^.

TRIM52 belongs to the C-V subfamily of TRIM proteins based on the structure of its C-terminal domains^3^. TRIM52 is unique among the TRIM family members because it contains only the RING domain and a B-Box domain. More intriguingly, the RING domain of TRIM52 is the largest among the classical RING domains; thus, TRIM52 is considered as an non-canonical antiviral TRIM protein^19^. However, TRIM52 does not exhibit any antiviral activity against lentiviruses^19^. In addition, the function of TRIM52 is limited.

This study demonstrates that human TRIM52 possesses antiviral activity against JEV replication. The anti-JEV activity of TRIM52 is manifested in its capacity to inhibit virus production and to repress the expression level of viral protein upon transient overexpression of TRIM52 in both BHK-21 and 293T cells. In addition, we found that TRIM52 interacted with NS2A and induced NS2A ubiquitination, resulting in NS2A degradation. Finally, we found that the E3 ubiquitin ligase activity of TRIM52 is essential for its anti-JEV activity.

## Results

### Ectopic expression of TRIM52 restricts JEV replication

**Figure 1:**
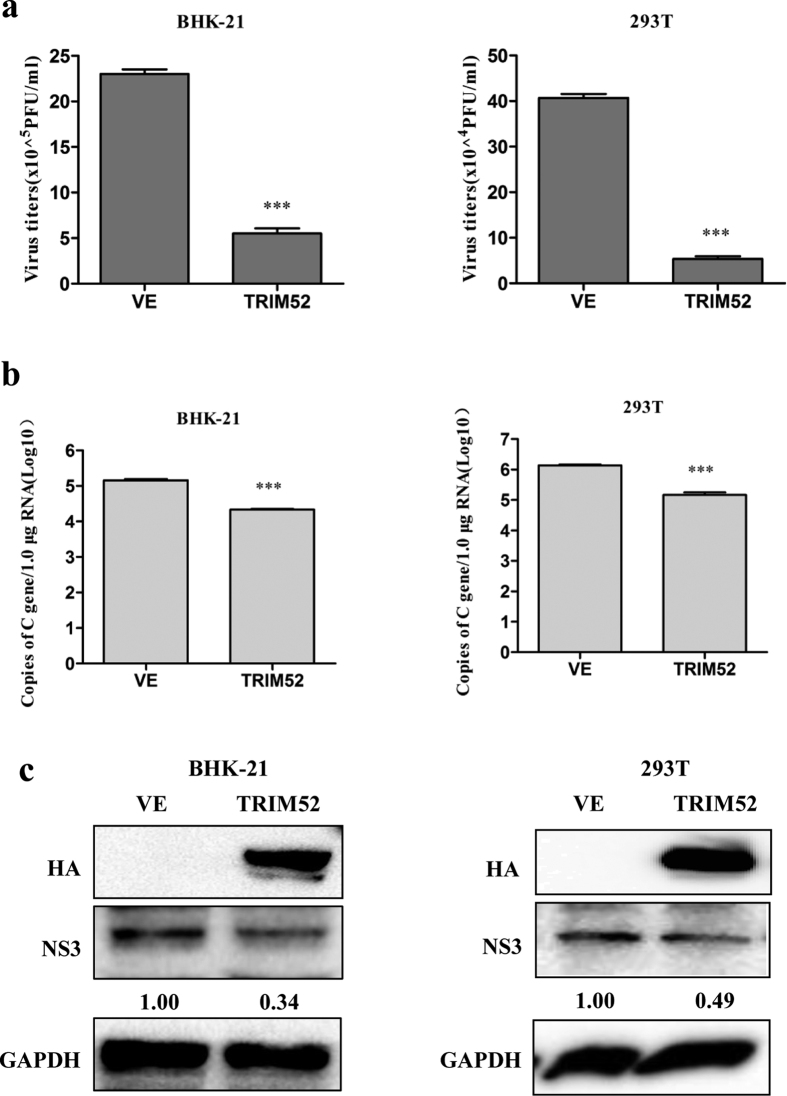
TRIM52 expression impairs JEV infection in BHK-21 and 293T cells. The BHK-21 or 293T cells in 12-well plates were transfected with 2 μg of TRIM52 expression plasmids or with empty vector for 30 h. The cells were subsequently infected with JEV at an MOI of 1. The cells and supernatants were collected at 24 h post-infection. Indicated experiments were performed to evaluate the antiviral activity of TRIM52 against JEV infection. (**a**) Production of progeny virus in culture supernatants of BHK-21 (left) and 293T cells (right) was measured by plaque assay. (**b**) Absolute quantification of intracellular JEV C gene copies in BHK-21 (left) and 293T cells (right) through real-time PCR analysis. (**c**) Western blot analysis of the protein level of JEV NS3 in BHK-21 (left) and 293T cells (right) by using the indicated antibodies. GAPDH served as loading control at an equal sample loading. Three asterisks indicate that a statistical difference exists between empty vector and TRIM52 expression cells at P < 0.001.

Many members of the TRIM family act as regulator in host–virus interaction; they either positively or negatively impact virus replication, as well as directly or indirectly restrict virus replication. This study investigated the potential antiviral function of TRIM52 against JEV infection. To test whether TRIM52 can influence JEV replication, we transfected BHK-21 and 293T cells with plasmids expressing HA-tagged TRIM52 or with an empty vector as control. The control and TRIM52–overexpressing cells were both infected with JEV at an MOI of 1. The culture supernatants were harvested 24 hour post-infection (hpi), and the titers of the progeny virus were determined using plaque forming unit assay on BHK-21 cells. Figure 1a shows that TRIM52 overexpression reduced JEV titers both in BHK-21 (left panel) and 293T cells (right panel). Moreover, gene copies of JEV C gene and protein level of NS3 in infected cells were detected, demonstrating the antiviral activity of TRIM52 against JEV. As shown in Fig. 1b, the intracellular C gene levels of JEV were significantly reduced in TRIM52-overexpressing cells compared with that in the vector control cells. In addition, Western blot analysis indicated that the NS3 protein levels of JEV were significantly repressed in the presence of TRIM52. These results suggest that TRIM52 exerted antiviral activity against JEV replication both in BHK-21 and 293T cells.

### TRIM52 interacts with NS2A

**Figure 2:**
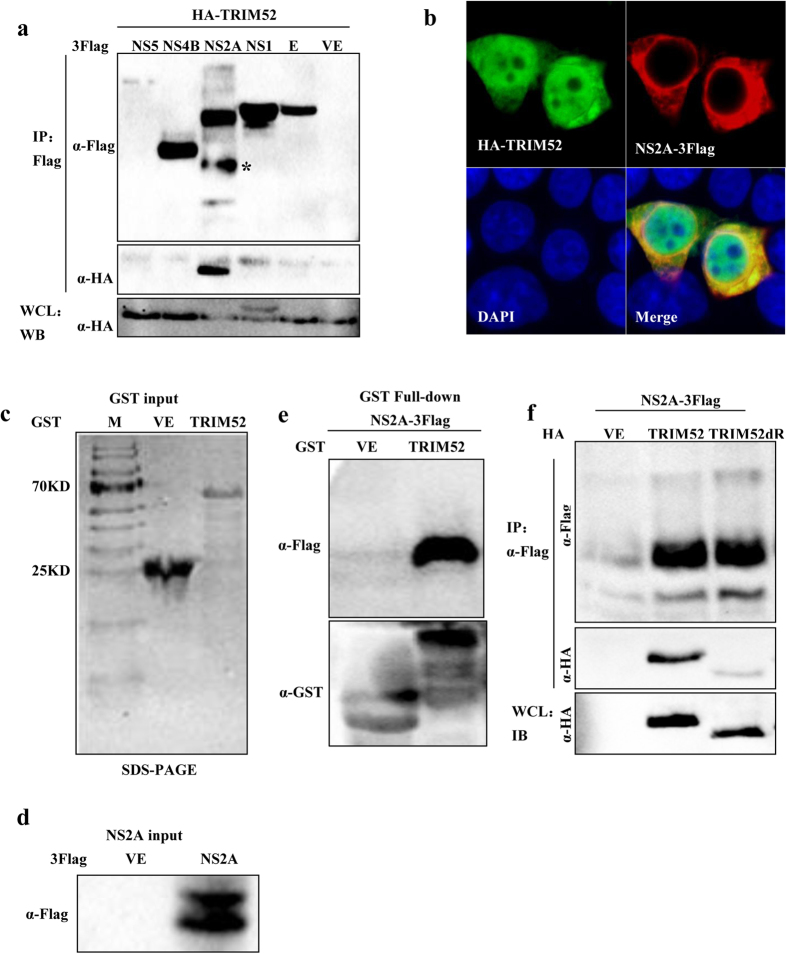
Interaction of TRIM52 with JEV NS2A protein. (**a**) 293T cells in six-well plates were co-transfected with expression plasmids encoding HA-TRIM52 plus the indicated plasmids expressing Flag-tagged JEV viral proteins. The cells were harvested at 36 hpt, and the cell lysates were prepared for Western blot analysis using the indicated antibodies. Immunoprecipitation (IP) was performed using anti-Flag affinity gel. WCL, whole cell lysates. (**b**) Co-localization of TRIM52 with NS2A. 293T cells were co-transfected with plasmids expressing HA-TRIM52 plus NS2A-Flag. The cells were fixed at 24 hpt and were subjected to immunofluorescence to detect HA-TRIM52 (green) and NS2A-Flag (red). The nuclei were stained with DAPI (blue). Images were obtained using confocal microscope (Carl Zeiss MicroImaging, Inc.). (**c**) Purified proteins of GST and GST-TRIM52 were analyzed by SDS-PAGE. (**d**) Western blot analysis of the anti-Flag affinity NS2A from 293T-NS2A cell lysates. (**e**) GST pull-down and Western blot analysis of the interaction between TRIM52 and NS2A. (**f**) IP using anti-Flag affinity gel and Western blot analysis for the interaction of NS2A with TRIM52 and RING domain deleted TRIM52 in 293T cells co-transfected with NS2A-Flag or with empty vector plus HA-TRIM52 and HA-TRIM52dR.

Studies have reported that JEV infection blocks interferon (IFN) induced Janus kinase (JAK) signal transducer and activation of transcription (STAT) signaling pathway to evade the antiviral actions of IFN^20^,^21^. We thus speculated that TRIM52 interacts with certain JEV viral proteins, restricting JEV replication. Co-immunoprecipitation (IP)-Western blot analysis was used to detect the interaction between TRIM52 and JEV viral proteins. The plasmids expressing individual Flag-tagged JEV proteins were co-transfected with HA-tagged TRIM52. The results shown in Fig. 2a indicates that NS2Ainteracts with TRIM52. Furthermore, confocal microscopy results indicated that TRIM52 co-localized with NS2A in the cytosol (Fig. 2b).

To verify the direct interaction of TRIM52 with NS2A, we performed GST pull-down analysis by using purified GST-tagged TRIM52 and GST protein control. However, we failed to express JEV NS2A protein in prokaryotic system; thus, anti-flag affinity NS2A proteins from 293T cell lysates were used in the GST pull-down assays. The results showed that GST-TRIM52 but not GST protein could capture NS2A (Fig. 2d,e). Moreover, we investigated the interaction between NS2A and RING domain-deleted TRIM52. Figure 2f shows that the interaction between TRIM52 and NS2A was attenuated after deletion of the RING domain. These results suggest that TRIM52 interacted with JEV viral NS2A via a direct physical interaction.

### TRIM52 self-ubiquitylation in 293T cells

**Figure 3:**
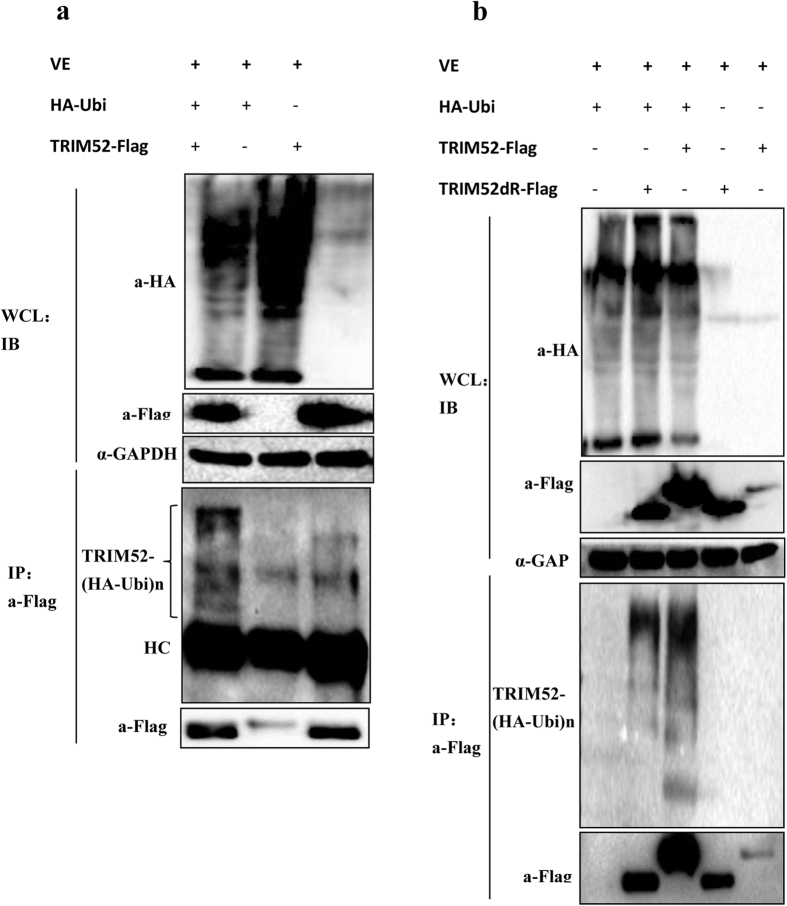
*In vivo* self-ubiquitination of TRIM52. (**a**) Plasmids expressing Flag-tagged TRIM52 or empty vector were co-transfected with or without HA-tagged ubiquitin in 293T cells. The cells were harvested at 30 hpt, and then subjected to an *in vivo* ubiquitination assay. (**b**) The 293T cells were co-transfected with Flag-tagged full length TRIM52, RING domain deleted TRIM52 (TRIM52-dR), and empty vector with or without HA-tagged ubiquitin. The cells were collected at 30 hpt for ubiquitination assay. HC, heavy chain of antibody.

We performed an *in vivo* ubiquitylation assay in 293T cells to evaluate the E3 ubiquitin ligase activity of the RING finger domain of TRIM52. The 293T cells were co-transfected with Flag-tagged TRIM52 with or without HA-tagged ubiquitin. The cells were collected at 30 hpt; subsequently, the cell lysate was precipitated with anti-Flag affinity gel, and then analyzed by Western blot using the indicated antibodies to detect ubiquitylated TRIM52. Figure 3a shows that poly-ubiquitylated TRIM52 was detected only when Flag-tagged TRIM52 was co-expressed with HA-tagged ubiquitin.

The E3 ligase activity of most TRIM proteins is RING domain dependent. We then investigated the importance of RING domain to the E3 ligase activity of TRIM52. As shown in Fig. 3b, compared with that of the full-length TRIM52, the smear signals in TRIM52 RING domain-deleted mutant significantly decreased. These results suggest that TRIM52 self-ubiquitylated in 293T cells. Moreover, the RING domain of TRIM52 is essential for its E3 ligase activity. These results also suggest that TRIM52 mediated the ubiquitylation of its interaction proteins via a RING-type E3 ligase activity.

### Overexpression of TRIM52 mediates NS2A degradation via proteasome pathway

**Figure 4:**
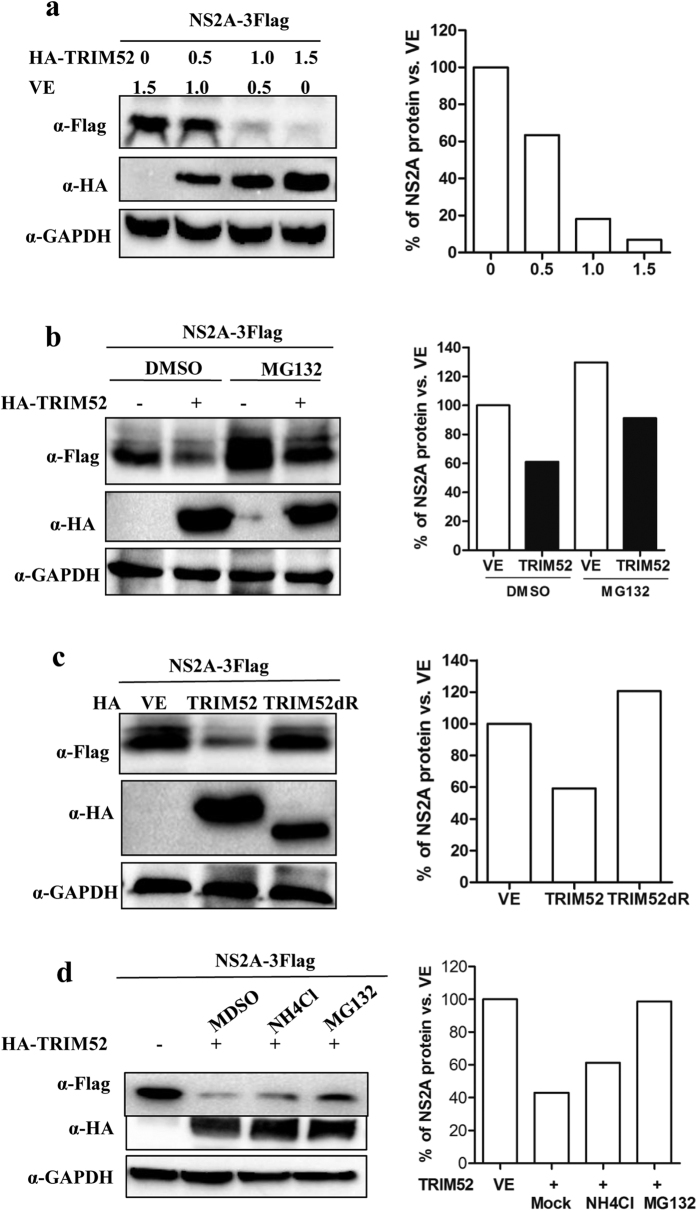
TRIM52 mediates NS2A degradation of NS2A. (**a**) Co-transfection of 293T cells with a fixed amount of NS2A-expressing plasmid and with increasing amounts of TRIM52-encoding plasmid, empty vector was used to equalize the doses of DNA plasmid. The cells were collected at 30 hpt, and the protein levels of TRIM52, and NS2A were evaluated through Western blot analysis. GAPDH was used as normalizer, and relative quantification of detected signal was analyzed using Image J software (right panel). (**b**) The 293T cells were co-transfected with 50 ng of NS2A-expressing plasmid plus 2000 ng of TRIM52-encoding plasmid or with empty vector. MG132 or DMSO was added at 24 hpt, and the cells were collected after 10 h of treatment. The protein levels of TRIM52, and NS2A were evaluated through Western blot analysis. GAPDH was used as normalizer, and relative quantification of detected signal was analyzed using Image J software (right panel). (**c**) The 293T cells were co-transfected with 50 ng of NS2A-expressing plasmid plus 2000 ng of wild type or RING domain lacking TRIM52-encoding plasmid or with empty vector. The cells were collected at 30 hpt, and the protein levels of TRIM52, and NS2A were evaluated by Western blot analysis. GAPDH was used as normalizer, and relative quantification of detected signal was analyzed using Image J software (right panel). (**d**) The 293T cells were co-transfected with 50 ng of NS2A-expressing plasmid plus 2000 ng of TRIM52-encoding plasmid or with empty vector. MG132, NH4Cl (20 mM) or DMSO was added at 24 hpt, and the cells were collected after 10 h of treatment. The protein levels of TRIM52, and NS2A were evaluated through Western blot analysis. GAPDH was used as normalizer, and relative quantification of detected signal was analyzed using Image J software (right panel).

TRIM52 interacted with JEV NS2A, and an *in vivo* self-ubiquitylation assay indicated that TRIM52 demonstrated E3 ligase activity. We thus investigated whether TRIM52 could influence the stability of NS2A protein. For this purpose, 293T cells were co-transfected with a fixed amount of Flag-tagged NS2A-expressing plasmid (50 ng) and with increasing amounts of HA-tagged TRIM52-encoding plasmids (500, 1000, and 2000 ng), equalizing the DNA doses with empty vector. The cells were harvested at 30 hpt, and the expression of NS2A was analyzed by Western blot. Figure 4a shows that an increasing dose of TRIM52 reduced the NS2A protein level. We subsequently, evaluated whether TRIM52 mediated NS2A protein degradation via the proteasome-dependent pathway. We transfected 293T cells with a fixed amount of NS2A-expressing plasmids (50 ng) plus TRIM52-expressing plasmid (2000 ng) or with empty vector control. The proteasome inhibitor MG132 (10 μM) or dimethyl sulphoxide (DMSO) was added at 24 hpt, and the cells were collected after 10 h. Figure 4b shows that NS2A protein levels significantly decreased in the presence of TRIM52 without MG132 treatment (Fig. 4b, right side, black bars). By contrast, NS2A protein levels were rescued by MG132 treatment.

The RING domain was essential for the E3 ligase activity of TRIM52. We thus investigated whether TRIM52-mediated NS2A protein degradation was dependent on the RING domain. We co-transfected 293T cells with a fixed amount of plasmid encoding NS2A (50 ng) plus 2000 ng of full-length plasmid encoding TRIM52 or TRIM52 mutant that lacks the N-terminal RING domain (TRIM52dR). After 30 h, the cells were collected, and NS2A expression levels were analyzed by Western blot analysis. Similar results were observed (Fig. 4c), that is, NS2A protein levels were significantly repressed in cells transfected with full-length TRIM52 but not in cells transfected with RING domain deleted mutant. In addition, we also investigated the contribution of lysosome degradation pathway on TRIM52-mediated degradation of NS2A. As shown in Fig. 4d, we did not observe any rescue of NS2A in the presence of NH4Cl (20 mM, an inhibitor of lysosome acidification), whereas MG132 restored NS2A protein level to DMSO and NH4Cl treatment group. Overall, these results indicate that TRIM52 could mediate NS2A protein degradation, and the RING domain is essential for this function. Moreover, TRIM52-meidated degradation of NS2A is proteasome pathway dependent.

### TRIM52 promotes ubiquitination of NS2A protein

**Figure 5:**
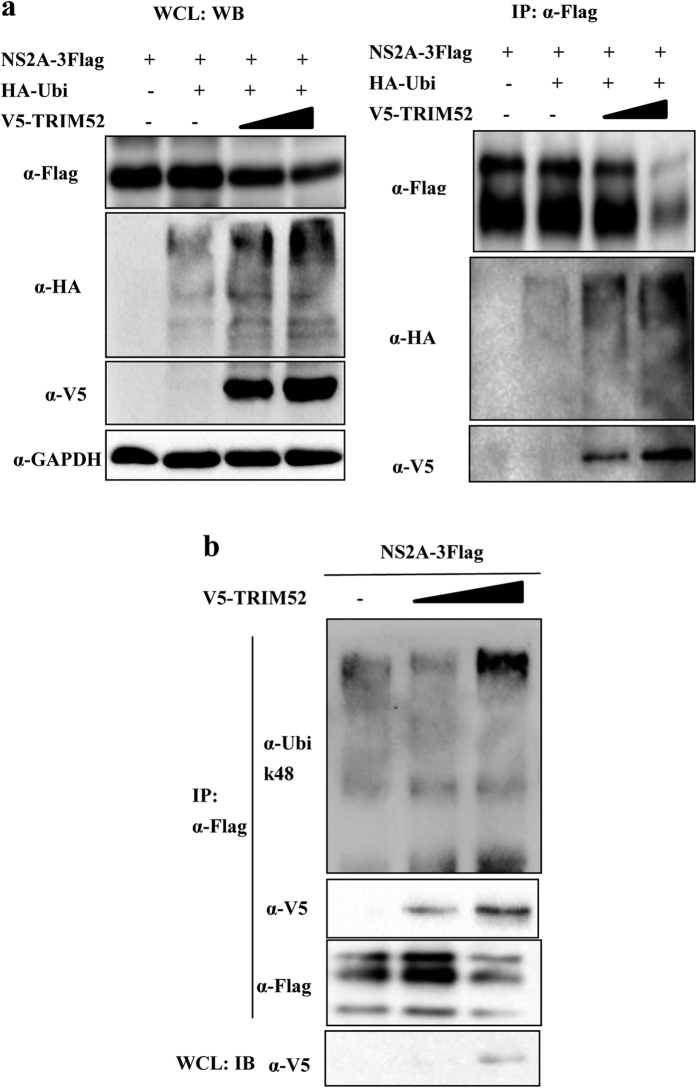
TRIM52 induces ubiquitination of NS2A. (**a**) NS2A-Flag (2000 ng) was co-expressed with HA-Ubi (1000 ng) and with increasing amounts of TRIM52-V5 (500 ng, 2000 ng) in 293T cells. The cells were harvested at 30 hpt, and IP was performed using anti-Flag affinity gel. The precipitates were analyzed through Western blot analysis using anti-Flag, anti-HA, anti-V5 and anti-GAPDH antibodies. (**b**) Co-transfection of NS2A-Flag (2000 ng) and increasing amounts of TRIM52-V5 (500 ng, 2000 ng) in 293T cells. The cells were collected at 30 hpt, and IP was performed using anti-Flag affinity gel. The precipitates were analyzed through Western blot analysis using anti-Flag, anti-V5 and anti-K48-ubiquitin antibodies.

We evaluated whether TRIM52 can mediate ubiquitination of NS2A protein. First, 293T cells were co-transfected with plasmid encoding NS2A-Flag plus V5-TRIM52 or with empty vector control in the presence or absence of HA-ubi. After 30 hpt, the cells were harvested and an IP was performed using anti-Flag affinity gel to analyze the ubiquitination of NS2A protein. Figure 5a shows that when NS2A-Flag was co-expressed with V5-TRIM52 in the presence of HA-ubi, smear signals were significantly increased in a TRIM52 dose-dependent manner. In addition, we determined the effect of TRIM52 on the K48-linked ubiquitination of NS2A. We co-transfected 293T cells with NS2A-Flag plus V5-TRIM52 or with vector control. The cells were collected at 30 hpt, and NS2A from cell lysates was precipitated using anti-Flag affinity gel. Western blot assay was subsequently performed using the indicated antibodies. Figure 5c shows that overexpression of TRIM52 significantly increased the K48-linked ubiquitination of NS2A. These results demonstrate that TRIM52 expression promotes NS2A ubiquitination.

### RING domain is critical for the antiviral function of TRIM52

**Figure 6:**
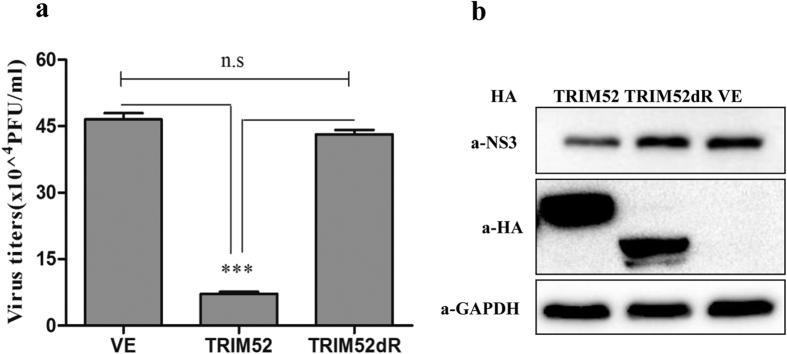
RING domain of TRIM52 is required for its antiviral activity against JEV replication. The 293T cells in 12-well plates were transfected with plasmid expressing full length TRIM52, RING domain-deleted TRIM52 and empty vector for 30 h and then infected with JEV (MOI = 1). The cells and culture supernatants were collected at 24 hpi. (**a**) Production of progeny virus in supernatants was determined using plaques assay. (**b**) Intracellular JEV NS3 protein was detected via Western blot analysis using the indicated antibodies. GAPDH was used to normalize the total protein loading. ***P < 0.001.

TRIM52 is a RING-type E3 ligase and it can mediate NS2A degradation via the ubiquitin-proteasome pathway. We thus speculated that the RING domain of TRIM52 also contributes to the antiviral activity of TRIM52. To validate this conjecture, we transfected 293T cells with plasmids encoding either the full-length TRIM52or RING domain deleted TRIM52 (TRIM52dR) or with empty vector 30 h prior to JEV infection at an MOI of 1. Progeny virus in culture supernatants was collected at 24 hpi, and viral titers were determined by plaque assay. The cells with full-length TRIM52 showed an obviously reduced viral production compared with the cells with TRIM52dR and empty vector control (Fig. 6a). By contrast, viral production in cells with TRIM52dR versus empty vector control did not vary (Fig. 6a). Moreover, JEV NS3 protein expression was significantly reduced in cells overexpressing the full-length TRIM52 compared with cells overexpressing TRIM52dR and vector control (Fig. 6b). No difference was observed between cells with TRIM52dR and empty vector (Fig. 6b). Overall, these results suggest that the RING domain of TRIM52 is required for TRIM52 to control JEV replication.

## Discussion

An increasing number of studies have regarded TRIM proteins as cancer-related factors^22^,^23^,^24^,^25^,^26^ and host antiviral factors^14^,^15^,^27^. Most TRIM proteins exhibit a broad spectrum of antiviral effects. TRIM22 is endowed with a potent capacity to repress various viral infections, including HIV^28^, HBV^16^,^29^, EMCV^30^, and IAV^17^. Li’s group has reported that TRIM56 exhibits a broad antiviral activity against bovine viral diarrhea virus (BVDV)^31^, yellow fever virus (YFV), dengue virus serotype 2 (DENV2), human coronavirus virus (HCoV)^32^, and influenza A and B viruses^33^. These TRIM proteins are among the host antiviral restriction factors. They directly target viral proteins to restrict virus replication. In addition, many members of the TRIM family proteins positively regulate host innate immunity response to develop host defense^34^. Ubiquitinated TRIM26 promotes TBK1 to interact with NEMO, resulting in the expression of type I IFNs^15^. Upon infection with viral DNA or RNA, TRIM9 short isoform undergoes Lys-63-linked auto-polyubiquitination and recruits GSK3β to TBK1, resulting in the activation of the IRF3 signaling pathway^14^.

Currently, few TRIM proteins have been reported that they involved the interaction between host and flavivirus. TRIM79α (also known as TRIM30D or TRIM30-3) presents only in rodent, which was the first reported and identified as an INF-induced TRIM protein that serviced as a flaviviurs restriction factor^35^. TRIM79α directly targeted NS5 from Langat virus (LGTV) and Tick-borne encephalitis virus (TBEV) for degradation through lysosomes pathway to restrict virus replication. However, TRIM79α did not target NS5 from West Nile virus (WNV), nor could it inhibit WNV replication. What’s more, TRIM79α is an essential mediator for IFN response specific to TBEV infection. TRIM56 is a virus- and IFN-inducible host antiviral factor. Liu and colleagues firstly demonstrated that human TRIM56 exhibited direct antiviral activity against YFV and DENV by targeting intracellular viral RNA replication^32^. Although the detail antiviral mechanisms of TRIM56 restricting YFV and DENV remaining unknown. The E3 ligase activity and integrity of the C-terminus of TRIM56 is essential for its antiviral activity against YFV and DENV. In addition, TRIM21 has been demonstrated that it as a negative regulator of IFNβ production mediated by IRF3 during JEV infection in human microglial cells (CHME3)^36^. Thus, JEV could exploit TRIM21 to attenuate host antiviral innate immune response during the early stage of infection.

TRIM52 is an unusual member of the TRIM family proteins because it contains an expanded RING domain and lacks the putative PRY/SPRY (B30.2) domain^19^. Little is known about the function of TRIM52. TRIM52 is a novel and non-canonical antiviral TRIM gene, although it does not exhibit antiviral function against lentiviruses^19^. Uchil reported that TRIM52 positively regulates NF-κB transcription activity^10^, although the mechanism remains unknown. We also found that TRIM52 could activate NF-κB transcription activity, and induce the expression of TNFα and IL-6. However, the expression TRIM52 could not increase the protein level of phosphorylated p65. Microscopy results indicated that TRIM52 was distributed in the cytosol and nucleus (Unpublished data).

Among the flavivirus NS proteins, NS2A protein is a small, hydrophobic transmembrane protein that is involved in virus life cycle and subversion of antiviral responses^37^,^38^,^39^,^40^. Dengue virus NS2A blocks the IFN-β induced nuclear translocation of STAT1-p^40^. The 13th alanine in KUNV NS2A is essential to antagonize IFN response and viral virulence^41^. JEV NS2A specifically inhibits double-stranded RNA-activated protein kinase PKR mediated antiviral response^39^. Currently, few studies have reported on the interaction between flavivirus NS2A and cellular proteins possibly because of the lesser significance of NS2A in flavivirus life cycle compared with other NS proteins, such as, NS3 and NS5. Studying the interaction between flavivirus NS2A and host cellular proteins will elucidate the viral replication and will provide a novel strategy to develop antiviral drugs.

This study demonstrates that ectopic overexpression of TRIM52 inhibits JEV replication both in BHK-21 and 293T cells (Fig. 1). Few TRIM proteins, including TRIM22^17^,^42^, TRIM25^43^ and TRIM79α^44^, directly exert antiviral activity. TRIM52 also directly interacted with JEV NS2A protein (Fig. 2). Moreover, TRIM52 mediated the ubiquitination of JEV NS2A to be degraded via the proteasome-dependent pathway. As shown in Fig. 4b, the protein level of NS2A was repressed in the presence of TRIM52 along with DMSO treatment. Addition of proteasome inhibitor MG132 obviously restored NS2A protein level. Moreover, the results in Fig. 3 indicate that the RING domain of TRIM52 was essential for its E3 ubiquitin ligase activity. The RING domain-deleted TRIM52 mutant lost its capacity to degrade NS2A proteins (Fig. 4c), resulting in truncation did not restrict JEV replication (Fig. 6). In summary, our data demonstrated that TRIM52 exerted antiviral activity against JEV replication by targeting the viral NS2A protein.

## Materials and Methods

### Cell lines and viruses

Baby hamster kidney BHK-21 cells (BHK-21; ATCC, CCL-10) and human embryonic kidney 293T cells (HEK293T; ATCC, CRL-11268) were grown in Dulbecco’s modified essential medium (DMEM; Invitrogen, USA) containing 10% fetal bovine serum (Gibco), 100 U/ml penicillin (GENVIEW) and 10 μg/ml streptomycin sulfate (GENVIEW) at 37 °C in a humidified 5% CO2 incubator. The JEV SX09S-01 strain, a genotype group I virus^45^, was used in this study, and virus titer assay was conducted in BHK-21 cells.

### Antibodies, reagents, and plasmids

Various commercially available antibodies were used in this study. Mouse monoclonal antibodies against Flag-tag and HA-tag, rabbit polyclonal antibodies against Flag-tag, Cy3 or FITC-conjugated goat anti-mouse antibodies, and FITC-conjugated goat anti-rabbit antibodies were purchased from ABclonal Biotech Co., Ltd (USA). Horseradish peroxidase-conjugated (HRP) goat anti-mouse and goat anti-rabbit IgG (H + L) secondary antibodies were obtained from Boster Bioengineering Ltd (China). Rabbit anti-JEV NS3 polyclonal antibodies and EasyBlot anti mouse IgG (HRP) were acquired from GeneTex Inc (USA). Mouse anti-GAPDH, anti-GST and anti-His monoclonal antibodies were purchased from Proteintech Group Inc. (USA). Rabbit anti-ubiquitin (linkage-specific K48) was purchased from Abcam (Cambridge, MA). Anti-Flag M2 affinity gel, dimethyl sulphoxide (DMSO), NH4Cl, and proteasome inhibitor MG132 were obtained from Sigma (St. louis, MO).

cDNA encoding full-length NS1, NS2A, NS4B, and NS5 from JEV^45^ were cloned into mammalian expression vector pCR3.1/3Flag. cDNA-encoding full length human TRIM52 or TRIM52 mutant was cloned into pCR3.1/3Flag or pCAGGS-HA vector. For pull-down experiment, the full lengths of TRIM52 and TRIM52 RING domain truncation (TRIM52dR) were cloned into the prokaryotic expression vector pGEX-4T-1. All constructs were identified through sequencing.

### Viral infection and virus titer determination

BHK-21 or 293T cells in 12-well plates were transfected with indicated plasmids. The cells were infected with JEV at an MOI of 1 at 30 hpt. The cells and supernatants were collected at 24 hpi. The progeny virus titers were measured using plaque forming unit (PFU) assay. Confluent monolayers of BHK-21 cells were infected with 10-fold serially diluted JEV samples in 12-well plates. After 1 h of incubation, 2% methylcellulose overlay was added. The plaques were visualized 4 days (d) post-infection (p.i.) by fixing with 10% formaldehyde and staining with crystal violet. The amount of plaques were observed and statistically analyzed. All virus titers were expressed as PFU/milliliter (PFU/ml).

### Real-time quantitative RT-PCR

Total RNA was extracted with the TRIzol reagent (Invitrogen, Grand Island, NY, USA) according to the manufacturer’s instructions. Total RNA (1.0 μg) was reverse transcribed using First Strand cDNA Synthesis Kit (TOYOBO) according to the manufacturer’s instructions. The mRNA levels of JEV C genes were determined through relative quantitative real-time PCR by using SYBR Green Real-time PCR Master Mix (TOYOBO), and fluorescent signals were analyzed by an ABI StepOne Plus system (Applied Biosystems). All reactions were performed in triplicate and the mRNA level of the housekeeping gene glyceraldehyde-3-phosphate dehydrogenase (GAPDH) was used as endogenous reference control.

### Preparation of recombinant bacterial proteins

TRIM52-GST and TRIM52dR-GST were expressed in *Escherichia coli* BL21 (DE3) strain induced with 0.5 mM isopropyl-1-thio-β-D-galactopyranoside (IPTG) at 16 °C for 18 h. GST-tagged TRIM52 and TRIM52dR were purified with glutathione sepharose 4B beads (GE Healthcare Life Science) according to the manufacturer’s protocol.

### Immunoblotting and IP

Cells were collected and treated with lysis buffer containing 1.19% HEPES, 0.88% NaCl, 0.04% EDTA, 1% NP40 and a protease inhibitor (Roche, UK). The protein concentration of whole-cell lysates was determined using bicinchoninic acid protein assay kit (Thermo Scientific) to evaluate protein expression. Equal amounts of proteins were separated using 12% sodium dodecyl sulfate polyacrylamide gels (SDS-PAGE) and transferred onto polyvinylidene fluoride (PVDF) membranes (Roche, UK). The membranes were blocked with 5% non-fat milk in 1× Tris-buffered saline (TBS) with 5% Tween-20 (DGBio, Beijing, China) for 4 h at room temperature (RT). The membranes were subsequently incubated with diluted primary antibodies at RT or at 4 °C for 2 or 16 h. Anti-rabbit or anti-mouse IgG antibodies conjugated to HRP were used as secondary antibodies. An enhanced chemiluminescence substrate was used in detection using HRP kit (Thermo Scientific, USA). All immunoblots images were performed using Bio-Rad ChemiDoc XRS+ instrument and image software.

For IP, the cells were lysed with lysis buffer containing protease inhibitors. Samples were subjected to centrifugation for 10 min at 4 °C to remove cellular debris. Cell lysates were incubated with anti-Flag M2 affinity gel in rolling incubator at 4 °C overnight. Lysates were discarded after a brief centrifugation at 3,000 g for 5 min at 4 °C. The beads were washed five times with cold lysis buffer prior to elution by incubation at 95 °C in 1× sample buffer (62.5 mM TRIS [pH 6.8], 10% glycerol, 15 mM EDTA, 4% 2-ME, 2% SDS, and bromophenol blue).

### Immunofluorescence

Cells were fixed in 4% paraformaldehyde for 20 min and permeabilized with 0.1% Triton X-100 at RT for 10 min. The cells were washed three times with phosphate-buffered saline (PBS) and then incubated with rabbit anti-Flag and mouse anti-HA antibody for 2 h. After three washes with PBS, the cells were incubated with FITC-conjugated goat anti-rabbit antibodies and Cy3-conjugated goat anti-mouse antibodies for 1 h. The cells were washed again three times with PBS and then incubated with 4′,6-diamidino-2-phenyl-indole (DAPI, Sigma) for 5 min. Fluorescent images were obtained using a confocal laser scanning microscope (LSM 510 Meta; Carl Zeiss).

### Statistical analysis

All experiments were performed in triplicate. GraphPad Prism software Version 5 (GraphPad Prism Version 5, GraphPad Software, La Jolla, CA, USA, 2012) was used in this study. The various treatments were compared using an unpaired, two-tailed Student’s t-test with an assumption of unequal variance. P < 0.05 was considered statistically significant. In addition, P < 0.01 and P < 0.001 were marked with two (**) and three (***) asterisks, respectively.

## Additional Information

**How to cite this article**: Fan, W. *et al*. TRIM52 inhibits Japanese Encephalitis Virus replication by degrading the viral NS2A. *Sci. Rep.* **6**, 33698; doi: 10.1038/srep33698 (2016).
